# Real-World Assessment of Personalized Approach With Voglibose Fixed-Dose Combination in Type 2 Diabetes Mellitus

**DOI:** 10.7759/cureus.57494

**Published:** 2024-04-03

**Authors:** Nirmal Parmar, Ajay Kumar Gupta, Kunal Jhaveri, Balachandran A, Gaurav Chhaya, Sandeep Kansara, Rathish Nair, Krishnaprasad R Korukonda

**Affiliations:** 1 Department of Internal Medicine, Prisha Medical Care, Ahmedabad, IND; 2 Department of Internal Medicine, Madhumeha Clinic, New Delhi, IND; 3 Department of Internal Medicine, Vishesh Clinic of Internal Medicine, Ahmedabad, IND; 4 Department of Diabetology, ABC Medical Centre, Erode, IND; 5 Department of Internal Medicine, Shivam Medi Care Clinic, Ahmedabad, IND; 6 Department of Endocrinology, Dr. Sandeep Kansara's Diabetes, Thyroid, and Hormone Clinic, Udaipur, IND; 7 Department of Medical Strategic Affairs, Torrent Pharmaceuticals Ltd., Ahmedabad, IND

**Keywords:** fixed-dose combination, ppbg, fbg, hba1c, trivoglitor, type 2 diabetes mellitus

## Abstract

Background: Post-prandial hyperglycemia (PPHG) remains a complex aspect in the management of type 2 diabetes mellitus (T2DM) in the Indian population due to uncertainty in the optimal utilization of alpha-glucosidase inhibitors (AGIs) either as standalone therapy or in combination, whether initiated initially or as a sequential therapy.

Methods: This was a post-approval, observational, multicentric clinical study conducted at 50 centers according to principles of the International Council for Harmonisation of Technical Requirements of Pharmaceuticals for Human Use Guideline for Good Clinical Practice (ICH-GCP) and Declaration of Helsinki and local ethics approval. Descriptive and analytical statistics were applied for conclusion and categorical variables using SPSS version 29.0.1.0 (171) (Armonk, NY: IBM Corp.).

Results: Protocol analyses of 515 cases revealed baseline demographics as follows: age 57.35±10.04 years, weight 72.86±10.92 kg, and BMI 28.33±6.07 kg/m^2^. Comorbidities included hypertension (N=169, 32.82%), thyroid disorders (N=99, 19.22%), and heart failure (N=92, 17.86%). Concomitant oral antidiabetics (OADs) prescribed as DPP4i (9.50%), SGLT2i (19.20%), and DPP4i+SGLT2i (7.20%). Study drug reduced glycosylated hemoglobin (HbA1c) by 13.77% (1.25% mean change, p<0.01), fasting blood glucose (FBG) by 23.69% (44.61 mg/dL mean change, p<0.01), post-prandial blood glucose (PPBG) by 24.57% (70.46 mg/dL mean change, p<0.01), and body weight by 4.43% (3.21 kg mean change, p<0.01) over 12 weeks. A total of 161 patients accomplished targeted PPBG of <180 mg/dL (119.13 mg/dL mean change, p<0.01). Regression analysis considering PPBG and HbA1c ≤7.5% showed a weak correlation between them (R-value=0.13, R-squared value=0.02), whereas between PPBG and HbA1c ≤9% yielded moderate positive correlation (R-value=0.53, R-squared value=0.28). There were no adverse events reported or analyzed during the observation period.

Conclusion: Voglibose fixed-dose combination (FDC) demonstrates significant effectiveness at the initial dosage when started early in the management of T2DM and high PPBG levels. Moreover, it exhibits good tolerability, thereby contributing to higher compliance among Indian patients who consume a high-carbohydrate diet.

## Introduction

Type 2 diabetes mellitus (T2DM) is a major chronic metabolic disorder in which there is increased glucose levels in the blood and affects a huge amount of the population globally with about 1.6 million deaths every year [1-3]. It is the most common form of diabetes and accounts for over 90% of all diabetes mellitus cases, a condition which is characterized by diminished insulin secretion by β-cells of the pancreas, inadequate compensatory insulin secretion, and insulin resistance (IR) [4,5]. When managing T2DM, post-prandial blood glucose (PPBG) is a crucial factor that requires aggressive control as it plays a significant role in determining glycated hemoglobin (HbA1c) levels. Reducing PPBG levels can substantially lower HbA1c levels in patients with T2DM [6,7]. Scientific studies have revealed that PPBG stands as an independent risk factor for cardiovascular diseases and is a reliable predictor of both cardiovascular and all-cause mortality. Cardiovascular diseases stand as a major cause of mortality in individuals affected with T2DM, contributing to 75-80% of the total mortality rate [8,9]. Thus, it is of great importance to manage the levels of PPBG to achieve adequate glycemic control [10].

Previous Indian studies substantiate the evidence for the use of fixed-dose combination (FDC) in more than 50% of study patients [11]. Fixed-dose combinations (FDCs) help in reducing pill burden and frequency of dosing, thereby, improving patient adherence to the drug therapy [12,13]. The general principles of recommended care in the Research Society for the Study of Diabetes in India (RSSDI) guidelines also mention the use of triple therapy as a patient-centric approach, if the glycemic targets are not achieved with two agents [14]. A one-hour post-load plasma glucose value of ≥8.6 mmol/L has been associated with a greater risk of progression to diabetes [15,16]. RSSDI recommends alpha-glucosidase inhibitors (AGIs) as one of the oral agents to be started as triple therapy. Following the widespread acceptance of dual therapy of OADs, a triple-drug combination of glimepiride, metformin, and voglibose was introduced, considering their individual clinical benefits as well as their synergistic action [17]. Real-world studies present extensive datasets sourced from diverse patient populations and offer valuable insights into the long-term safety and effectiveness of a drug [18]. The triple drug combination of metformin, glimepiride, and voglibose has been shown to be effective in the control of glycemic pentad in Indian patients. This triple FDC demonstrated efficacy in controlling both fasting and PPBG levels and thereby, regulating HbA1c and glycemic variations [19]. The present study was conducted to assess real-world clinical assessment on clinical safety and efficacy data of triple-drug FDC of glimepiride, metformin, and voglibose in patients with T2DM.

## Materials and methods

Ethical conduct of the study

This study was conducted in accordance with principles of the Declaration of Helsinki (Brazil, October 2013), Good Clinical Practice for clinical research in India, 2005, New Drugs and Clinical Trials Rules 2019, The International Council for Harmonisation of Technical Requirements of Pharmaceuticals for Human Use Guideline for Good Clinical Practice E6 (R2), and Indian Council of Medical Research’s National Ethical Guidelines for Biomedical and Health Research involving Human Participants, 2017. The study was approved by the Sangini Hospital Ethics Committee (EC registration number: IORG0007258). The study was also registered with the clinical trials registry of India (#CTRI/2023/06/053828). All the enrolled subjects received a Patient Information Sheet which included trial-specific essential information like description and purpose of study, treatment options, and information on patient confidentiality and rights.

Study design and patient population

This was a post-approval, real-world clinical study that was observational in nature. The data were collected retrospectively from multiple centers. The study included 515 diabetic patients who sought treatment at various outpatient clinics across India, offering a diverse patient pool.

Subjects were selected based on inclusion and exclusion criteria. From the overall obtained data, patients who lacked baseline demographics or follow-up assessments (including age, gender, FBG, HbA1c, and PPBG) were not considered in the analyses and the final sample size was determined based on the available data. Included were the individuals of both genders, aged 18 years and above, diagnosed with uncontrolled T2DM and exhibited HbA1c levels equal to or exceeding 7%, FBG levels greater than 100 mg/dL, and PPBG levels equal to or surpassing 200 mg/dL. Eligible individuals had previously undergone treatment with FDC of glimepiride, metformin sustained release (SR), and voglibose at any dosage strength. Individuals with incomplete data or those deemed unsuitable for the study based on the investigator’s judgment due to specific conditions were excluded from participation. The study was conducted from June 2023 to September 2023.

The primary objectives of the study include assessment of the reduction of HbA1c, PPBG, and fasting blood glucose (FBG) levels along with overall body weight after four and 12 weeks of treatment; to discover the impact of PPBG reduction on HbA1c levels; to calculate responder rate of patients with targeted PPBG <180 and <160 mg/dL. The secondary objective includes the evaluation of the overall safety of the triple-drug FDC at the end of the 12-week study period.

Study procedure

The study comprised three assessment phases. Activities at visit 1 (screening and enrolment) included patient enrolment, comprehensive medical history recording, demographic data collection, and laboratory tests like HbA1c, PPBG, and FBG. Visit 2, held after four weeks, aimed to analyze modifications in FBG, PPBG, and body weight. Finally, visit 3, scheduled at the 12-week mark, focused on evaluating patient global assessment (PGA) scores, alterations in HbA1c levels, and changes in body weight. Complete information, encompassing demographic factors such as gender, age, weight, height, any concurrent health conditions, PGA score, as well as medical and surgical history, was meticulously documented for each patient in the case report form. Furthermore, essential laboratory findings, including PPBG, HbA1c, and FBG, were consistently recorded within the case record form. Treatment compliance was assessed using case record forms. Patients were considered to have completed the study if they were followed up and adhered to the 12-week treatment period, which was documented in the case record form. During the entire study duration, close monitoring was done to capture any possible treatment-emergent adverse events.

Study drug

The study drug was a fixed dose combination of glimepiride, metformin, and voglibose available in the following four different strengths: (1) glimepiride (1 mg) + metformin (500 mg) + voglibose (0.2 mg); (2) glimepiride (1 mg) + metformin (500 mg) + voglibose (0.3 mg); (3) glimepiride (2 mg) + metformin (500 mg) + voglibose (0.2 mg); (4) glimepiride (2 mg) + metformin (500 mg) + voglibose (0.3 mg). The drug was meant to be administered orally and swallowed as a whole without chewing. The frequency of tablet ingestion was determined by treating physician.

Statistical methods

All statistical analyses were performed using SPSS version 29.0.1.0 (171) (Armonk, NY: IBM Corp.). Descriptive statistics was used to analyze quantitative variables like patient demographics, change in PPBG, HbA1c, FBG, and body weight from baseline and expressed as mean, median, standard deviations (SD), change from baseline (CFB), CFB%, minimum and maximum values with 95% confidence interval. Frequency and percentage were reported for qualitative variables. The significance of continuous variables was assessed using the Student’s paired t-test, employing a two-tailed test, and considering a p-value <0.05 as statistically significant. Regression analysis was employed to analyze the association of PPBG with HbA1c, with results presented as R-value (Pearson correlation coefficient) and R-squared value. A total of 515 cases were analyzed for various clinical, behavioral, or investigation characteristics for a likely prevalence of at least 1-10%.

## Results

Patient demographics and baseline characteristics

**Figure 1:**
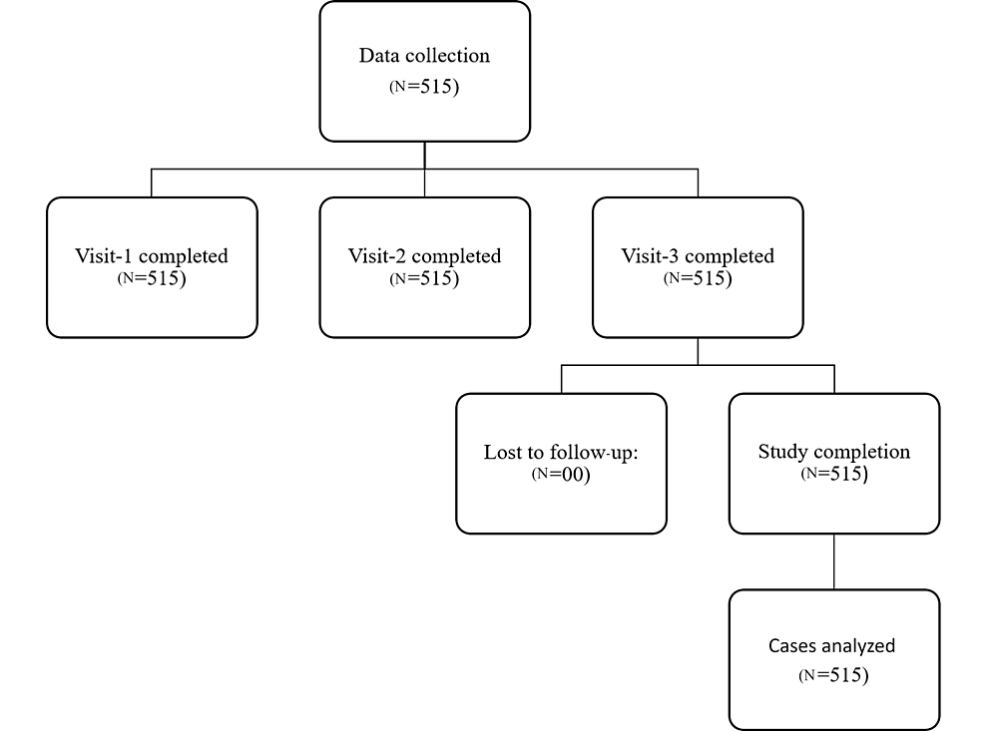
Disposition of the study participants across the study duration.

The disposition of the study participants has been depicted in Figure 1. The study included 515 T2DM patients (441 males and 74 females) with a mean age of 57.35±10.04 years, weight of 72.86±10.92 kg, and body mass index (BMI) 28.33±6.07 kg/m^2^. Other baseline characteristics included comorbidities like hypertension (N=169, 32.82%), followed by thyroid disorders (N=99, 19.22%), heart failure (N=92, 17.86%), and various other medical conditions. In terms of addiction, 505 patients exhibited one or more addictions, with the majority of patients addicted to smoking (N=134, 26.02%), closely followed by the habit of alcohol consumption (N=128, 24.85%), combination of smoking and alcohol consumption (N=117, 22.72%), and various other addictions (Table 1).

**Table 1:** Demographic distribution and baseline characteristics of study participants. N=number of subjects

Parameters		Total (N=515)
Demographics	Age (years) (mean, SD)	57.35 (10.04)
	Height (cm) (mean, SD)	162.36 (16.23)
	Weight (kg) (mean, SD)	72.86 (10.92)
	Body mass index (kg/m^2^) (mean, SD)	28.33 (6.07)
	Gender (N, %)	Male (441, 85.63%)
		Female (74, 14.37%)
Concomitant antidiabetics prescribed (N, %)	Sodium-glucose cotransporter-2 inhibitors	99, 19.20%
	Insulin	87, 16.90%
	Voglibose, dipeptidyl peptidase 4 inhibitors	67, 13.00%
	Dipeptidyl peptidase 4 inhibitors	49, 9.50%
	Sulfonylureas	45, 8.70%
	Glucagon-like peptide-1 receptor agonists	42, 8.20%
	Sodium-glucose cotransporter-2 inhibitors, dipeptidyl peptidase 4 inhibitors	37, 7.20%
	Other antidiabetics	84, 16.31%
Comorbid condition (N, %)	Hypertension	169, 32.82%
	Thyroid disorders	99, 19.22%
	Heart Failure	92, 17.86%
	Gastrointestinal problem	71, 13.80%
	Asthma	58, 11.30%
	Anxiety	54, 10.50%
	Chronic obstructive pulmonary disease	53, 10.30%
Treatment pattern (N, %)	Glimepiride (1 mg) + metformin (500 mg) + voglibose (0.2 mg)	217, 42.10%
	Glimepiride (1 mg) + metformin (500 mg) + voglibose (0.3 mg)	138, 26.80%
	Glimepiride (2 mg) + metformin (500 mg) + voglibose (0.2 mg)	121, 23.50%
	Glimepiride (2 mg) + metformin (500 mg) + voglibose (0.3 mg)	39, 7.60%

Among 515 patients, 402 patients (78.06%) had a family history of T2DM while the rest did not have a family history of the same. The most prevailing factors that determined the initiation of triple-drug FDC were HbA1c control (N=201, 39.03%) and PPBG control (N=113, 21.95%). Most commonly prescribed concomitant antidiabetics were sodium-glucose cotransporter-2 inhibitors (SGLT2i) (N=99, 19.20%), insulin (N=87, 16.9%), both dipeptidyl peptidase 4 inhibitors (DPP4i) and voglibose (N=67, 13.0%), dipeptidyl peptidase 4 inhibitors (DPP4i) (N=49, 9.50%), and several other combinations of antidiabetics were prescribed.

Primary endpoint results

**Figure 2:**
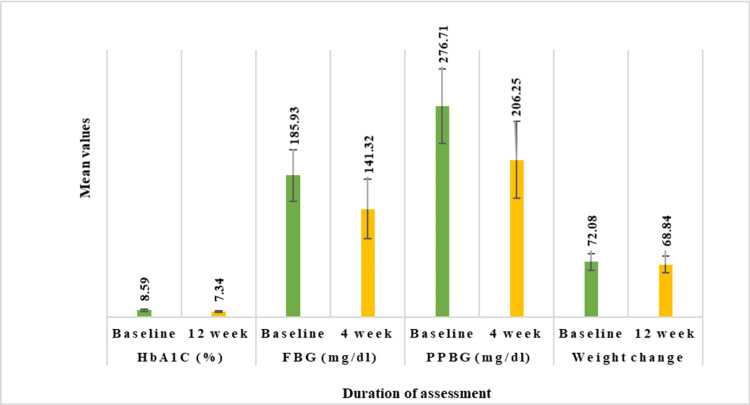
Impact of triple-drug FDC on HbA1c, FBG, PPBG, and body weight in all T2DM patients. N=515 (overall population). Values at four and 12 weeks are significant at p<0.01 (derived using paired t-test). FDC: fixed-dose combination; HbA1c: glycated hemoglobin; FBG: fasting blood glucose; PPBG: post-prandial blood glucose; T2DM: type 2 diabetes mellitus

**Figure 3:**
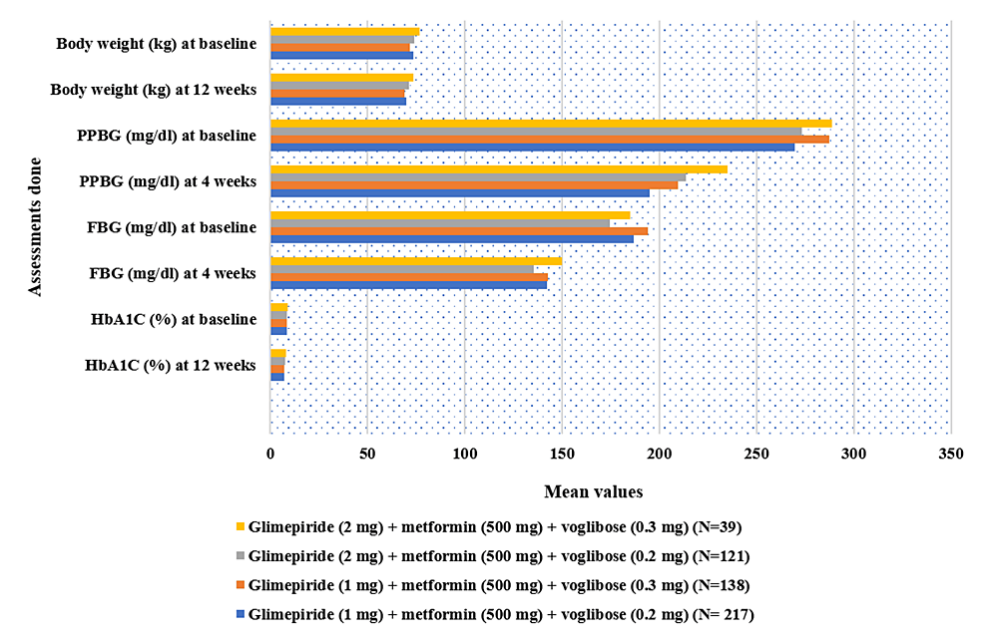
Impact of triple-drug FDC on HbA1c, FBG, PPBG, and body weight for different prescribed doses. Values at four and 12 weeks are significant at p<0.01 (derived using paired t-test) compared to baseline. N=39 [glimepiride (2 mg) + metformin (500 mg) + voglibose (0.3 mg)], N=121 [glimepiride (2 mg) + metformin (500 mg) + voglibose (0.2 mg)], N=138 [glimepiride (1 mg) + metformin (500 mg) + voglibose (0.3 mg)], N=217 [glimepiride (1 mg) + metformin (500 mg) + voglibose (0.2 mg)]. FDC: fixed-dose combination; HbA1c: glycated hemoglobin; FBG: fasting blood glucose; PPBG: post-prandial blood glucose

Post-treatment with the study drug in the overall population with uncontrolled T2DM, HbA1c demonstrated a significant decrease from baseline level of (mean±SD) 8.59±1.19% to 7.34±1.01% (CFB% mean: 13.77% reduction, p<0.01) at 12 weeks follow-up period. Similarly, PPBG levels lowered significantly from a baseline reading of 206.71±50.21 mg/dL to 206.25±50.21 mg/dL (24.57% reduction, p<0.01) post-four weeks of treatment. FBG decreased from 185.93±33.81 mg/dL to 141.32±39.11 mg/dL (23.69% reduction, p<0.01) after four weeks of treatment. Body weight also exhibited a significant reduction from 72.86±10.92 kg to 69.66±10.92 kg (4.43% reduction, p<0.01) post-12 weeks of treatment (Figure 2). Furthermore, the study drug at each of the four doses exhibited a significant decrease (p<0.01) in all evaluated parameters (Figure 3).

Regression analysis was performed to establish the association between PPBG and HbA1c, which yielded an R-value (Pearson correlation coefficient) of 0.63. The R-value indicated a strong positive linear correlation between PPBG and HbA1c stating that as values of PPBG decreased, HbA1c levels decreased as well. An R-squared value of 0.40 indicated that approximately 40% of variability in the HbA1c levels can be explained by changes in PPBG. Similarly, multivariate regression analysis between PPBG, HbA1c, and body weight yielded a strong positive correlation signifying a strong association between change in PPBG levels and its impact on both HbA1c levels and body weight (R-value=0.79, R-squared value=0.62). Further regression analysis between PPBG and HbA1c levels was done after dividing the overall population into groups having baseline HbA1c levels ≤7.5% and ≤9%. The group with HbA1c levels ≤7.5% showed a very weak correlation between the specified variables (R-value=0.13, R-squared value=0.02), whereas the group with HbA1c ≤9% yielded a moderate positive correlation between the said parameters (R-value=0.53, R-squared value=0.28) signifying the effective role of study drug in late T2DM. Overall, the results showed a significant association between PPBG and HbA1c, and between PPBG, HbA1c, and body weight suggesting that PPBG levels played a significant role in influencing both HbA1c levels and body weight.

Post four-week treatment, responder rate for patients achieving targeted PPBG levels revealed that 101 patients (19.61%) achieved targeted levels of <160 mg/dL with a reduction from baseline mean value of 283.06±43.07 mg/dL to 143.35±13.08 mg/dL (p<0.01). Similarly, 161 patients (31.26%) accomplished targeted PPBG level of <180 mg/dL showing reduction from baseline mean value of 274.04±47.76 mg/dL to 154.91±18.68 mg/dL (p<0.01).

Secondary endpoint result

In terms of safety, none of the enrolled patients reported any treatment-related adverse events throughout the study duration. This finding highlights good tolerability of the study drug in managing patients with uncontrolled T2DM, regardless of the presence of comorbidities.

Sub-group analyses

**Figure 4:**
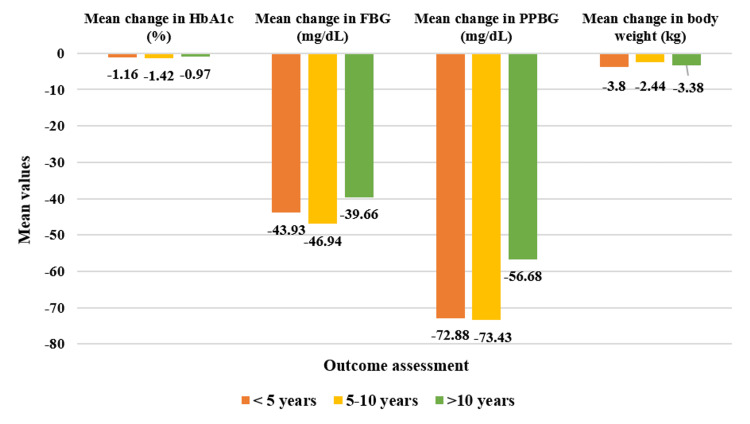
Impact of triple-drug FDC on HbA1c, FBG, PPBG, and body weight in various T2DM durations for overall population as represented by mean change from baseline. Duration of follow-up assessment from baseline: 12 weeks for HbA1c and body weight; duration of follow-up assessment from baseline: four weeks for FBG and PPBG, all values are significant at p<0.01 (derived using paired t-test), <5 years: N=193, 5-10 years: N=237, >10 years: N=85. N=515 (overall population). FDC: fixed-dose combination; HbA1c: glycated hemoglobin; FBG: fasting blood glucose; PPBG: post-prandial blood glucose

**Figure 5:**
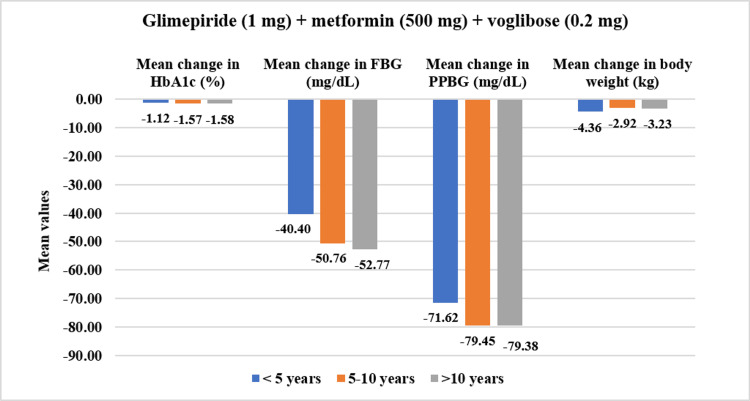
Impact of glimepiride (1 mg) + metformin (500 mg) + voglibose (0.2 mg) on HbA1c, FBG, PPBG, and body weight in various T2DM durations. Duration of follow-up assessment from baseline: 12 weeks for HbA1c and body weight; duration of follow-up assessment from baseline: four weeks for FBG and PPBG, all values are significant at p<0.01 (derived using paired t-test), <5 years: N=129, 5-10 years: N=75, >10 years: N=09 FDC: fixed-dose combination; HbA1c: glycated hemoglobin; FBG: fasting blood glucose; PPBG: post-prandial blood glucose; T2DM: type 2 diabetes mellitus

**Figure 6:**
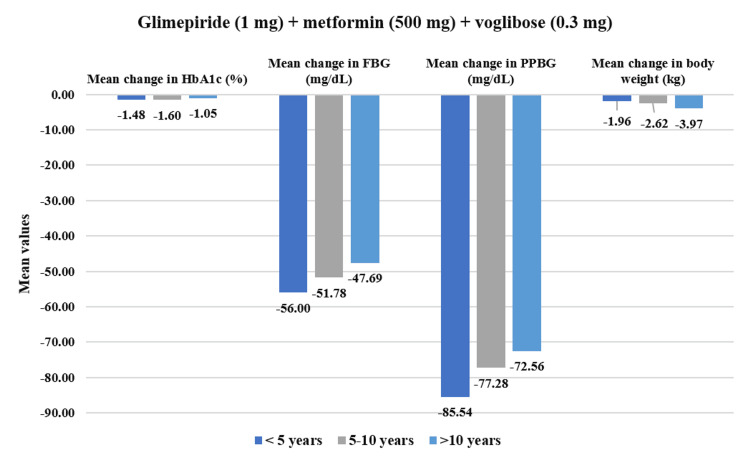
Impact of glimepiride (1 mg) + metformin (500 mg) + voglibose (0.3 mg) on HbA1c, FBG, PPBG, and body weight in various T2DM durations. Duration of follow-up assessment from baseline: 12 weeks for HbA1c and body weight; duration of follow-up assessment from baseline: four weeks for FBG and PPBG, all values are significant at p<0.01 (derived using paired t-test), <5 years: N=26, 5-10 years: N=80, >10 years: N=32. FDC: fixed-dose combination; HbA1c: glycated hemoglobin; FBG: fasting blood glucose; PPBG: post-prandial blood glucose; T2DM: type 2 diabetes mellitus

**Figure 7:**
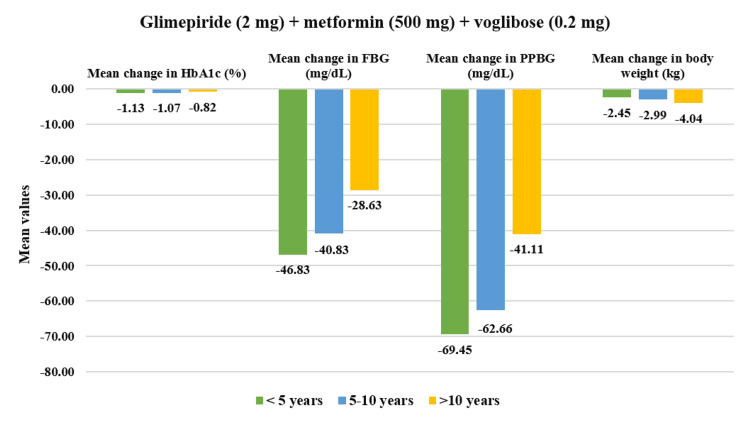
Impact of glimepiride (2 mg) + metformin (500 mg) + voglibose (0.2 mg) on HbA1c, FBG, PPBG, and body weight in various T2DM durations. Duration of follow-up assessment from baseline: 12 weeks for HbA1c and body weight; duration of follow-up assessment from baseline: four weeks for FBG and PPBG, all values are significant at p<0.01 (derived using paired t-test), <5 years: N=29, 5-10 years: N=65, >10 years: N=27. FDC: fixed-dose combination; HbA1c: glycated hemoglobin; FBG: fasting blood glucose; PPBG: post-prandial blood glucose; T2DM: type 2 diabetes mellitus

**Figure 8:**
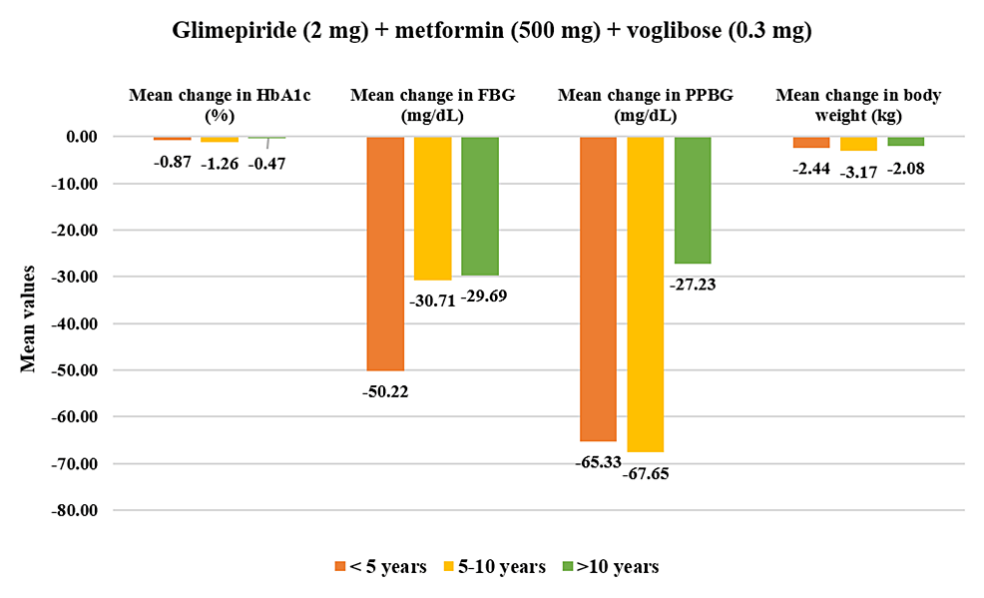
Impact of glimepiride (2 mg) + metformin (500 mg) + voglibose (0.3 mg) on HbA1c, FBG, PPBG, and body weight in various T2DM durations. Duration of follow-up assessment from baseline: 12 weeks for HbA1c and body weight; duration of follow-up assessment from baseline: four weeks for FBG and PPBG, values are significant at p<0.05 (derived using paired t-test), HbA1c (<5 years) and body weight 5-10 years) is not significant, <5 years: N=09, 5-10 years: N=17, >10 years: N=13. FDC: fixed-dose combination; HbA1c: glycated hemoglobin; FBG: fasting blood glucose; PPBG: post-prandial blood glucose; T2DM: type 2 diabetes mellitus

In the overall cohort, patients having a duration of T2DM <5 years (N=193) showed significant reductions in baseline HbA1c values by 12.64% (CFB%) (mean change: 8.50±1.38% to 7.34±1.04%, p<0.01). PPBG values showed a greater reduction of 25.88% (mean change: 275.41±44.96 mg/dL to 202.54±44.97 mg/dL, p<0.01). Similarly, FBG was reduced by 23.08% (mean change: 190.89±38.43 mg/dL to 146.96±44.49 mg/dL, p<0.01) while body weight was reduced by 4.75% (mean change: 73.22±9.97 kg to 69.72±9.78 kg, p<0.01). Moreover, for patients with T2DM duration of 5-10 years (N=237), HbA1c reduced significantly by 15.63% (mean change: 8.63±1.17% to 7.21±0.99%, p<0.01); PPBG reduced by 25.47% (mean change: 279.97±51.27 mg/dL to 206.54±55.35 mg/dL, p<0.01); FBG showed reduction by 25.23% (mean change: 183.25±31.56 mg/dL to 136.31±36.79 mg/dL, p<0.01), while body weight reduced by 4.12% (mean change: 71.95±12.04 kg to 69.09±12.28 kg, p<0.01). Finally, patients who have had T2DM for duration of >10 years (N=85) demonstrated a significant reduction in HbA1c by 11.11% (mean change: 8.66±0.61% to 7.69±0.93%, p<0.01), in PPBG by 19.07% (mean change: 270.55±52.74 mg/dL to 213.87±45.87 mg/dL, p<0.01), in FBG by 20.82% (mean change: 182.12±27.01 mg/dL to 142.46±29.75 mg/dL, p<0.01) and in body weight by 4.51% (mean change: 74.68±8.01 kg to 71.31±7.88 kg, p<0.01) (Figure 4). Moreover, the study drug demonstrated a significant decrease (p<0.01) across all four doses for varying durations of T2DM (Figures 5-8).

**Figure 9:**
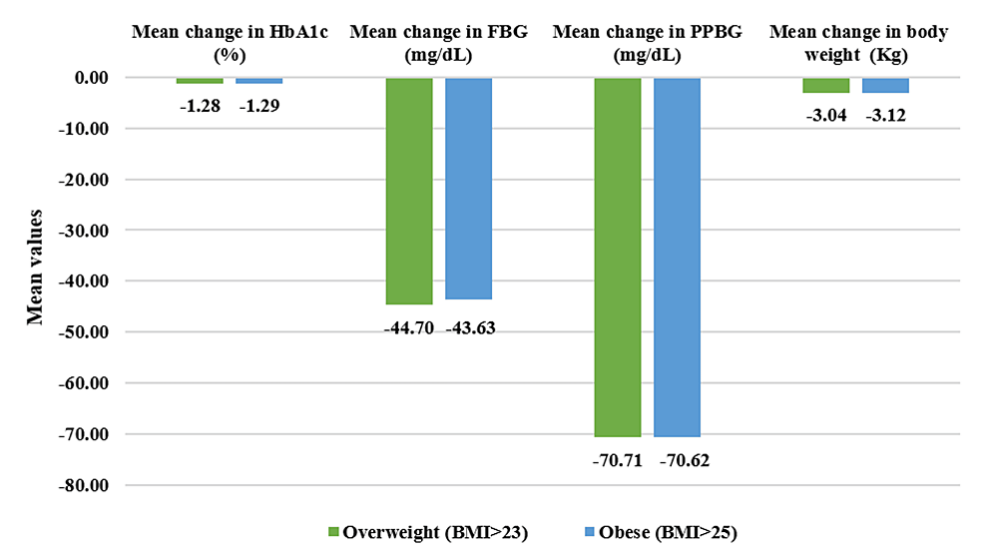
Effect of triple-drug FDC on HbA1c, FBG, PPBG, and body weight in overweight and obese patients in overall population as represented by mean change from baseline Duration of follow-up assessment from baseline: 12 weeks for HbA1c and body weight; duration of follow-up assessment from baseline: four weeks for FBG and PPBG, all values are significant at p<0.01 (derived using paired t-test). Overweight (BMI>23): N=469, obese (BMI>25): N=373, and N=515 (overall population). FDC: fixed-dose combination; HbA1c: glycated hemoglobin; FBG: fasting blood glucose; PPBG: post-prandial blood glucose

**Figure 10:**
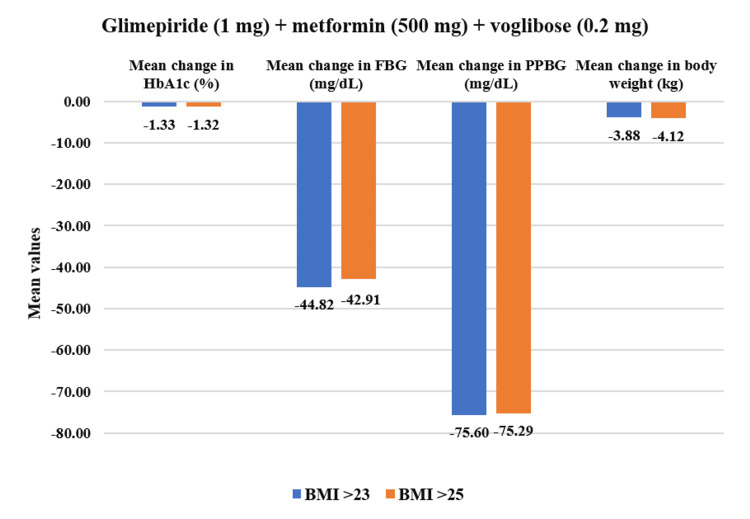
Impact of glimepiride (1 mg) + metformin (500 mg) + voglibose (0.2 mg) on HbA1c, FBG, PPBG, and body weight in overweight and obese patients. Duration of follow-up assessment from baseline: 12 weeks for HbA1c and body weight; duration of follow-up assessment from baseline: four weeks for FBG and PPBG, all values are significant at p<0.01 (derived using paired t-test). Overweight (BMI>23): N=192 and obese (BMI>25): N=152. FDC: fixed-dose combination; HbA1c: glycated hemoglobin; FBG: fasting blood glucose; PPBG: post-prandial blood glucose

**Figure 11:**
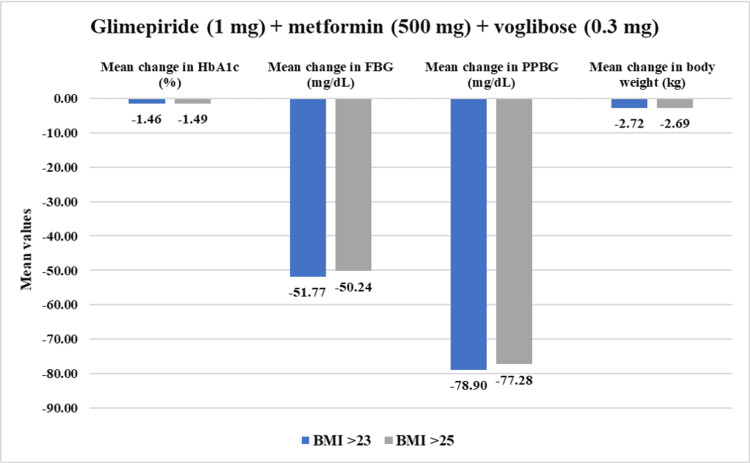
Impact of glimepiride (1 mg) + metformin (500 mg) + voglibose (0.3 mg) on HbA1c, FBG, PPBG, and body weight in overweight and obese patients. Duration of follow-up assessment from baseline: 12 weeks for HbA1c and body weight; duration of follow-up assessment from baseline: four weeks for FBG and PPBG, all values are significant at p<0.01 (derived using paired t-test). Overweight (BMI>23): N=132 and obese (BMI>25): N=100. FDC: fixed-dose combination; HbA1c: glycated hemoglobin; FBG: fasting blood glucose; PPBG: post-prandial blood glucose

**Figure 12:**
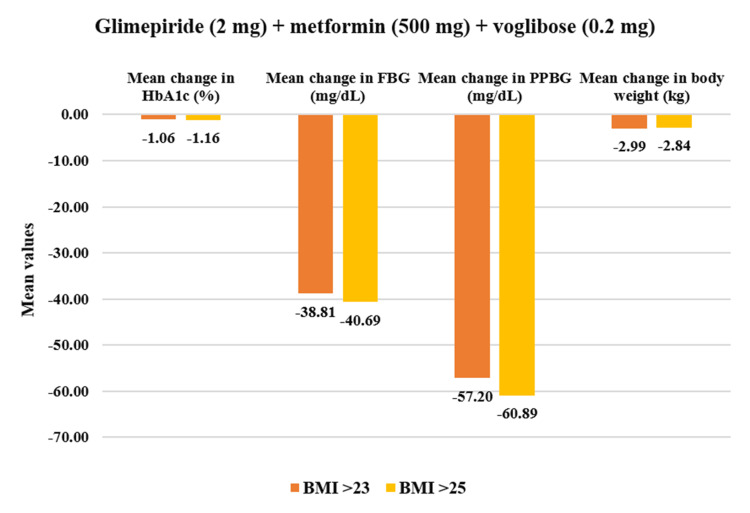
Impact of glimepiride (2 mg) + metformin (500 mg) + voglibose (0.2 mg) on HbA1c, FBG, PPBG, and body weight in overweight and obese patients. Duration of follow-up assessment from baseline: 12 weeks for HbA1c and body weight; duration of follow-up assessment from baseline: four weeks for FBG and PPBG, all values are significant at p<0.01 (derived using paired t-test). Overweight (BMI>23): N=107 and obese (BMI>25): N=89. FDC: fixed-dose combination; HbA1c: glycated hemoglobin; FBG: fasting blood glucose; PPBG: post-prandial blood glucose

**Figure 13:**
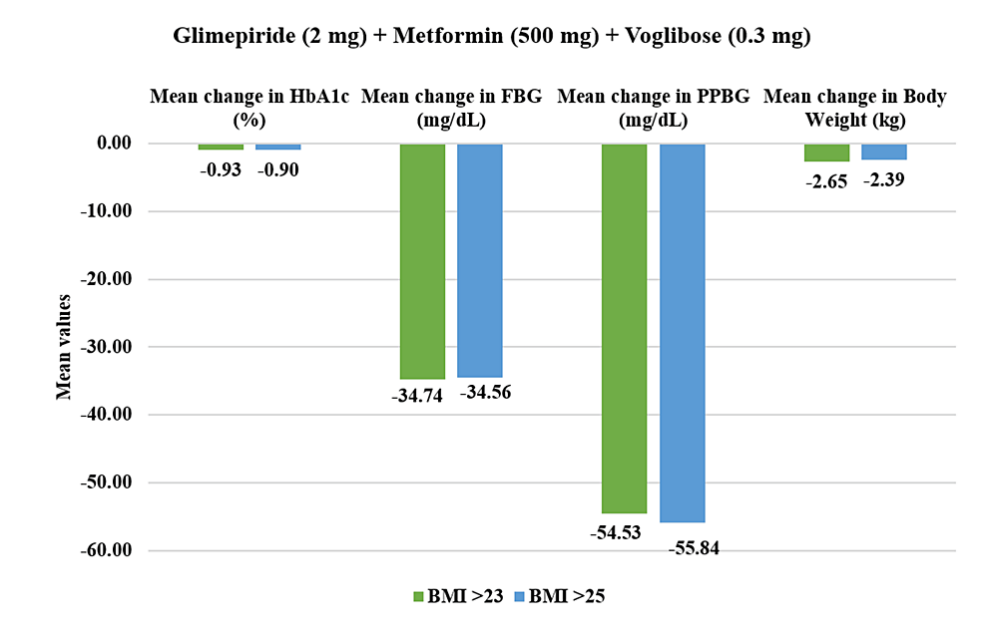
Impact of glimepiride (2 mg) + metformin (500 mg) + voglibose (0.3 mg) on HbA1c, FBG, PPBG, and body weight in overweight and obese patients. Duration of follow-up assessment from baseline: 12 weeks for HbA1c and body weight; duration of follow-up assessment from baseline: four weeks for FBG and PPBG, all values are significant at p<0.01 (derived using paired t-test). Overweight (BMI>23): N=38 and obese (BMI>25): N=32. FDC: fixed-dose combination; HbA1c: glycated hemoglobin; FBG: fasting blood glucose; PPBG: post-prandial blood glucose

From the overall population, overweight patients who had BMI >23 kg/m^2^ (n=469) demonstrated a statistically significant reduction in PPBG by 24.56% (mean value post-treatment: 207.11±51.54 mg/dL, p<0.01) and in FBG by 23.69% (141.26±39.34 mg/dL, p<0.01) after four weeks of treatment. Additionally, HbA1c reduced significantly by 14.05% (7.35±1.05%, p<0.01) while body weight reduced by 4.45% (70.10±11.03 kg) post 12 weeks of study drug usage. Within the obese population who had BMI >25 kg/m^2^ (n=373), PPBG reduced significantly by 24.53% (209.58±52.12 mg/dL, p<0.01) and FBG reduced by 23.33% (141.11±38.93 mg/dL, p<0.01). Moreover, HbA1c exhibited reduction by 14.31% (7.32±1.06%, p<0.01) while body weight showed reduction by 4.40% (70.14±11.66 kg, p<0.01) at the end of 12-week treatment period (Figure 9). Moreover, in all assessed parameters, the study drug showed a significant decrease (p<0.01) across all four doses for overweight and obese patients (Figures 10-13).

Results of PGA revealed that 241 (46.80%) patients had good results with the use of the study drug, followed by 145 (28.15%) patients who had very good results, 47 (9.13%) patients stated fair results and 82 (15.92%) patients stated excellent results in their health post study drug usage.

## Discussion

The present study effectively achieved its predetermined objectives, focusing on evaluating the safety and efficacy of the study drug in managing patients with uncontrolled T2DM with or without comorbidities. The combination of metformin, glimepiride, and voglibose stood as a well-tolerable and effective treatment in controlling HbA1c, PPBG, FBG, and body weight for effective management. Moreover, the drug showed positive results in obese (4.40% reduction) and overweight (4.45% reduction) patients for effective weight management post-12 weeks of usage.

Previous studies have shown the importance of PPBG control and its effect on HbA1c levels, stating that the reduction in PPBG levels significantly lowers HbA1c levels in T2DM patients. Controlling PPBG levels is a major factor in the management of T2DM [6]. Hence, timely management with the right drug proves to be beneficial in controlling the increased glycemic levels.

This study revealed a significant decrease in HbA1c and PPBG levels post-treatment with the study drug, aligning with a prior study's findings [19]. A study on triple-drug FDCs in managing T2DM reported effective achievement of glycemic control in a safe, well-tolerated, and cost-effective manner [17]. Rao and Faruqui similarly observed significant decreases in HbA1c, FBG, and PPBG levels in uncontrolled T2DM patients using a triple-drug combination of glimepiride, metformin, and voglibose [20].

Kalra et al. conducted a clinical study on 1365 diabetic patients (mean age 53.7±11.00 years) treated with voglibose [21]. At the end of the 12-week assessment period, there was a reduction in post-baseline mean HbA1c (7.29%), FBG (119.1 mg/dL), and PPBG (179.7 mg/dL). Body weight reduced by 1.00 kg. Jindal et al. revealed in their study that a combination of voglibose (0.2 mg taken thrice daily), glimepiride (2 mg taken twice daily), and metformin (500 mg taken twice daily) reduced baseline FBG (171.73±25.45 to 157.00±25.98, p<0.0001), PPBG (264.40±48.84 to 193.13±40.39, p<0.0001), and HbA1c (9.13±0.48 to 7.96±0.68, p<0.0001) after three months of treatment [22]. A retrospective study compared voglibose as an add-on therapy to sulfonylureas in Indian diabetic patients who were overweight and obese [23]. Voglibose significantly reduced HbA1c level after eight weeks (8.61±1.24 to 8.29±1.3, p<0.05) and after 12 weeks of treatment (8.61±1.24 to 7.53±1.25, p<0.01). The same study reported a significant reduction in body weight post-eight weeks (77.85±9.43 to 74.84±8.1 kg, p<0.05) and 12 weeks (77.85±9.43 to 73.52±7.68, p<0.05) of treatment. A parallel arm study by Murti et al. revealed that a combination of voglibose, glimepiride, and metformin was more effective compared to the dual glimepiride and metformin group in reducing baseline PPBG (266.88±21.68 mg/dL to 227.51±14.62, p<0.05), FBG (183.97±13.73 to 145.65±6.19, p<0.001), and HbA1c (8.19±0.35 to 7.60±0.28, p<0.005) after three months [24].

The outcomes of the present study are consistent with prior research investigations as discussed. Additionally, this study also established a positive correlation between PPBG, HbA1c, and body weight suggesting that PPBG levels play a significant role in influencing both HbA1c levels [25,26] and body weight, especially benefiting the patients having high HbA1c as well as PPBG and body weight [27,28]. PGA score stated that the study produced highly favorable outcomes, aligning precisely with the predefined aim and objectives. By acting as a competitive inhibitor of intestinal alpha-glucosidase, voglibose acts as a potent alpha-glucosidase inhibitor (AGI). It efficiently delays the digestion and absorption of dietary polysaccharides present in our diet, thereby enabling control over and reduction of both body weight and PPBG [6,29]. Across all the examined groups with varying durations of T2DM (<5, 5-10, and >10 years), the FDC effectively reduced HbA1c, PPBG levels, and body weight. These findings align with two studies where the combination of voglibose demonstrated reductions in HbA1c, PPBG, and body weight [6,30].

It is essential to acknowledge certain limitations associated with the study. This is an exploratory analysis of the personalized approach adopted by primary care physicians/specialists for the management of PPBG with a voglibose-based regimen. Therefore, well-structured appropriately designed controlled studies with long-term follow-up to offer insights into the drug's safety and effectiveness over extended periods of use will aid in further understanding of clinical effectiveness and safety of triple-drug FDC.

## Conclusions

This real-world study demonstrated that triple-drug FDC of glimepiride, metformin, and voglibose effectively reduced HbA1c, PPBG, FBG, and body weight in clinical cases of early-onset T2DM in the Indian population. Additionally, no treatment-related adverse events were observed despite the early initiation of voglibose in T2DM management.

## References

[1] Das AK, Wangnoo SK, Chawla R (2022). Expert consensus on triple combination of glimepiride, metformin, and voglibose usage in patients with type 2 diabetes mellitus in Indian settings. J Diabetol.

[2] Westman EC (2021). Type 2 diabetes mellitus: a pathophysiologic perspective. Front Nutr.

[3] Kharroubi AT, Darwish HM (2015). Diabetes mellitus: the epidemic of the century. World J Diabetes.

[4] Galicia-Garcia U, Benito-Vicente A, Jebari S (2020). Pathophysiology of type 2 diabetes mellitus. Int J Mol Sci.

[5] Gajera D, Trivedi V, Thaker P, Rathod M, Dharamsi A (2023). Detailed review on gestational diabetes mellitus with emphasis on pathophysiology, epidemiology, related risk factors, and its subsequent conversion to type 2 diabetes mellitus. Horm Metab Res.

[6] Dabhi AS, Bhatt NR, Shah MJ (2013). Voglibose: an alpha glucosidase inhibitor. J Clin Diagn Res.

[7] Yamasaki Y, Katakami N, Hayaishi-Okano R (2005). Alpha-glucosidase inhibitor reduces the progression of carotid intima-media thickness. Diabetes Res Clin Pract.

[8] Hershon KS, Hirsch BR, Odugbesan O (2019). Importance of postprandial glucose in relation to A1C and cardiovascular disease. Clin Diabetes.

[9] Hanssen NM, Kraakman MJ, Flynn MC, Nagareddy PR, Schalkwijk CG, Murphy AJ (2020). Postprandial glucose spikes, an important contributor to cardiovascular disease in diabetes?. Front Cardiovasc Med.

[10] Ivers NM, Jiang M, Alloo J, Singer A, Ngui D, Casey CG, Yu CH (2019). Diabetes Canada 2018 clinical practice guidelines: key messages for family physicians caring for patients living with type 2 diabetes. Can Fam Physician.

[11] Alam MS, Aqil M, Shah Qadry SA, Kapur P, Pillai KK (2014). Utilization pattern of oral hypoglycemic agents for diabetes mellitus type 2 patients attending out-patient department at a university hospital in New Delhi. Pharmacol Pharm.

[12] Böhm AK, Schneider U, Aberle J, Stargardt T (2021). Regimen simplification and medication adherence: fixed-dose versus loose-dose combination therapy for type 2 diabetes. PLoS One.

[13] Cazzola M, Matera MG (2017). Fixed-dose combination inhalers. Handb Exp Pharmacol.

[14] Chawla R, Madhu SV, Makkar BM, Ghosh S, Saboo B, Kalra S (2020). RSSDI-ESI clinical practice recommendations for the management of type 2 diabetes mellitus 2020. Indian J Endocrinol Metab.

[15] Bergman M, Chetrit A, Roth J, Jagannathan R, Sevick M, Dankner R (2016). One-hour post-load plasma glucose level during the OGTT predicts dysglycemia: observations from the 24year follow-up of the Israel Study of Glucose Intolerance, Obesity and Hypertension. Diabetes Res Clin Pract.

[16] Tricò D, Galderisi A, Mari A, Santoro N, Caprio S (2019). One-hour post-load plasma glucose predicts progression to prediabetes in a multi-ethnic cohort of obese youths. Diabetes Obes Metab.

[17] John M, Gopinath D, Kalra S (2015). Triple fixed drug combinations in type 2 diabetes. Indian J Endocrinol Metab.

[18] Blonde L, Khunti K, Harris SB, Meizinger C, Skolnik NS (2018). Interpretation and impact of real-world clinical data for the practicing clinician. Adv Ther.

[19] Faruqui AA (2016). Safety and efficacy of fixed dose combination of voglibose, glimepiride and metformin in Indian type 2 diabetes mellitus patients. Adv Diabetes Metab.

[20] Rao C, Faruqui AA (2013). Efficacy and safety of oral triple drug combination (voglibose, glimepiride and metformin) in the management of type 2 diabetes mellitus. Int J Curr Res Rev.

[21] Kalra S, Selvam AP, Shah AV (2020). Prospective multicenter observational study of voglibose in type 2 diabetes-victory. US Endocrinol.

[22] Jindal A, Jindal M, Kaur M, Kumar R, Brar RS (2014). Efficacy and safety of voglibose as an add-on triple drug in patients of type two diabetes mellitus uncontrolled with glimepiride and metformin in Punjabi population. Indian J Basic Appl Med Res.

[23] Talaviya PA, Saboo BD, Dodiya HG, Rao SK, Joshi SR, Modh VB, Ghadiya SV (2016). Retrospective comparison of voglibose or acarbose as an add-on therapy to sulfonylureas in Western Indian patients with uncontrolled overweight/obese type 2 diabetes. Diabetes Metab Syndr.

[24] Murti K, Sethi MK, Dey A, Lal CS, Pandey K, Das P (2016). Addition of voglibose to glimepiride and metformin have better glucose control in diabetics: a prospective, parallel-group and open-label comparative study. Int J Pharmacol.

[25] Haddadinezhad S, Ghazaleh N (2010). Relation of fasting and postprandial and plasma glucose with hemoglobinA1c in diabetics. Int J Diabetes Dev Ctries.

[26] Gupta S, Puppalwar PV, Chalak A (2014). Correlation of fasting and post meal plasma glucose level to increased HbA1c levels in type-2 diabetes mellitus. Int J Adv Med.

[27] Gummesson A, Nyman E, Knutsson M, Karpefors M (2017). Effect of weight reduction on glycated haemoglobin in weight loss trials in patients with type 2 diabetes. Diabetes Obes Metab.

[28] Kowsar R, Mansouri A (2022). Multi-level analysis reveals the association between diabetes, body mass index, and HbA1c in an Iraqi population. Sci Rep.

[29] Negishi M, Shimomura K, Proks P, Shimomura Y, Mori M (2008). Alpha glucosidase inhibitor voglibose can prevent pioglitazone-induced body weight gain in type 2 diabetic patients. Br J Clin Pharmacol.

[30] Kasthuri S, Poongothai S, Anjana RM (2021). Comparison of glycemic excursion using flash continuous glucose monitoring in patients with type 2 diabetes mellitus before and after treatment with voglibose. Diabetes Technol Ther.

